# The Proper Use of Fibre-Optic Sensors to Monitor the Condition of the Steam Boiler Hanger Rods

**DOI:** 10.3390/s23177522

**Published:** 2023-08-30

**Authors:** Magdalena Palacz, Bolesław Bąk, Łukasz Felkowski, Piotr Duda, Iliya Iliev

**Affiliations:** 1 Department of Production Engineering, Faculty of Organisation and Management, Silesian University of Technology, Akademicka 2A, 44-100 Gliwice, Poland; 2 Sumitomo SHI FW Energia Polska sp. z o.o., Młynarska 42, 01-171 Warszawa, Poland; boleslaw.bak@shi-g.com; 3 Institute of Thermal and Process Engineering, Cracow University of Technology, Al. Jana Pawła II 37, 31-864 Krakow, Poland; lukasz.felkowski@pk.edu.pl (Ł.F.); piotr.duda@pk.edu.pl (P.D.); 4 Department of Heat, Hydraulics and Environmental Engineering, “Angel Kanchev” University of Ruse, 7017 Ruse, Bulgaria; iki@uni-ruse.bg

**Keywords:** condition monitoring, hanger rod, FBG sensors

## Abstract

Fibre optic sensors with integrated Bragg gratings are widely used in the diagnostics of machinery and equipment. They achieved their popularity thanks to their relatively simple operating principles. In addition, they allow the continuous monitoring of several variable physical parameters of objects, such as strain or temperature change, which directly translates into immediate feedback regarding potential damage. However, despite the easy-to-understand operating principle, selecting a specific type for a particular application can be problematic. This article aims to present the process of selecting the optimal set of fibre-optic sensors with integrated Bragg grating, which can be used in the process of monitoring the stress state of hanger rods of an engineering object such as an industrial boiler. The hanger rods of such boilers require constant technical supervision; however, the current measurement methods do not provide an effective and non-invasive diagnostic method. Therefore, the authors have undertaken the task of developing a universal diagnostic strategy for hanger rods. To this end, they will present the results of an analysis of the applicability of FBGs, examples of the use of different types of sensors, their installation methods, and the technical capabilities of the equipment necessary to handle the signals recorded with these sensors. Exemplary results of strain measurements of a selected hanger rod performed by the traditional method used now and with a selected FBG fibre optic sensor will be presented. In conclusion, concrete technical suggestions will be presented to be implemented in the existing industrial facility during the next part of the study.

## 1. Introduction

Fibre Bragg Grating (FBG) sensors are optical sensors that use the unique spectral characteristics of light reflection from Bragg gratings in optical fibres. These sensors are used in a wide range of applications and industries, including structural health monitoring, civil engineering [1], aerospace and aviation [2], oil and gas [3], power generation, automation, and process control. The sensors can be installed in a variety of ways, including embedding them in materials, attaching them to surfaces, and placing them in contact with liquids. FBG sensors can also be integrated into wireless systems for remote monitoring and data collection.

Although the operating principle is relatively simple and the installation is rather easy, it has not yet been possible to find an application for FBG sensors to monitor the strain of hanger rods in industrial boilers. For this reason, the authors in this article, based on a review of literature sources and up-to-date technical solutions, present the most important aspects related to the preparation of the hardware measurement system for the selected technical application.

The motivation to address this topic is based on several facts. Firstly, the hanger rods of industrial boilers are axially loaded by design. However, the state of strain is different for each rod. Therefore, periodic measurements of the stress state of the bars are performed using frequency methods, assessing the level of the difference in the natural frequency of the subsequent rod. This method has certain advantages and disadvantages, the most important of which is the need to completely shut down the facility for the duration of the measurement [4].

Secondly, the boiler itself is a device that operates under varying loading conditions on the hanger rods—this generates constantly changing stress fields that cannot be measured when the object is turned off. Therefore, the idea for a health monitoring system that allows continuous measurement of the changing values of the stress range of each rod [5,6].

A third argument that motivates the topic is the existence of a potentially significant difference in the degree of stress of individual hanger rods, which is of great importance for the continuity of boiler operation. Continuous access to the results of measuring the varying stresses in the rods will allow an efficient response to situations that might pose a risk to operational safety.

A fourth reason that contributed to the topic presented in the article is the varying temperature field during the operation of an industrial boiler. For a measurement method based on modal analysis, the varying temperature field significantly affects the accuracy of the measurement, which can lead to an erroneous inference of results. In the case of the use of FBG sensors, there is no such danger, as it is now possible to use sensors that make it possible to determine the influence of the varying temperature field in relation to the change in the signal caused by a change in the deformation field of the rod in question. Another reason for addressing the topic described in this article is that FBG fibre-optic sensors allow the use of a single optical fibre and several Bragg gratings—a great advantage over classical sensors requiring a separate power supply. As is well known, fibre-optic sensors are immune to the effects of varying electromagnetic fields, and recent technical developments allow measurements in environments with an operating temperature of around 100 degrees Celsius.

A final motivating element is that the measuring system with FBG sensors is stable even over relatively long measuring distances—this fact is crucial in view of the dimensions of the hanger rods of industrial boilers.

Despite the extremely simple principle of fibre-optic sensors, the selection of a satisfactory solution for a specific research case can be problematic. The analysis of literature resources shows that no online system for measuring forces in rods in a thermal power boiler that is based on fibre-optic sensors has been developed so far. Therefore, the paper will present currently used methods for evaluating forces in bars and determining their accuracy. Next, an analysis of available solutions will be presented in terms of application in a power boiler. Finally, a project for a system for measuring forces in rods in a selected power boiler will be presented. The most important steps in the construction of such a diagnostic system based on fibre-optic sensors with a Bragg grating will be discussed. Exemplary results of strain measurements of a selected hanger rod performed by the traditional method used now and with a selected FBG fibre optic sensor will be presented.

In the conclusion of the introductory section, the authors will provide a brief overview of the overall structure of the article. In the following section, the basic features of FBG sensors will be discussed, highlighting those that are crucial for use in the measurement system of hanger rods in industrial boilers. This will be followed by a numerical model of the selected boiler with a detailed description of the stress pile-up points in the selected rods. The next section of the article will discuss the technical problems that are associated with the use of existing measurement methods. In the final part of the article, the authors present the proposed solution and discuss the resulting application possibilities. The last part includes conclusions, with a focus on the continuation of the research.

## 2. Types of FBG Sensors

FBGs are adaptable and lightweight strain and temperature sensors that may be multiplexed into sensor networks and integrated into a variety of material systems. FBG sensors have the advantages of being unaffected by electromagnetic interference, being able to measure multiaxis strain fields, and being able to assess local strain distributions for structural health monitoring applications. These benefits are accompanied by the costs of investment and data-gathering rate limitations for large networks as compared with electrical resistance or piezoelectric strain gauges.

**Figure 1:**
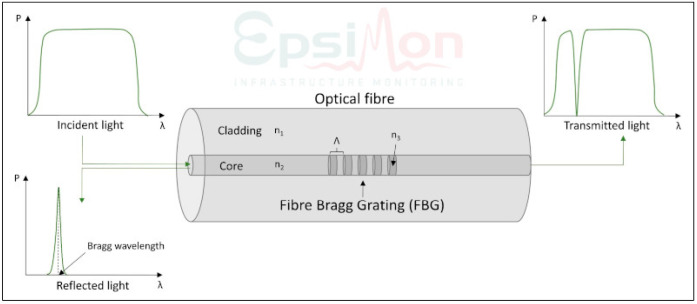
Spectral response and basic structure of a fibre Bragg grating (FBG) from EpsiMon. Reproduced with permission from Epsimon Ltd., Steeple Morden, UK. Copyright Epsimon Ltd. [7].

As presented in Figure 1, the periodic pattern in the fibre (i.e., FBG) reflects a specific wavelength of light, which depends on the periodicity of the grating. A change in periodicity, e.g., by lengthening the fibre at the location of the FBG, results in a shift in the reflected wavelength. Such an FBG is used to measure a wide variety of physical parameters, e.g., temperature, pressure, and strain, and is often used in harsh environments where conventional (electrical) sensors fail [7].

Among numerous types of FBG sensors, the most popular are the low-reflectivity sensors, where reflectivity depends strictly on factors like grating length, refractive index, and grating strength (<20% of peak reflection factor). As reflectivity has an important impact on the signal-to-noise ratio (SNR—defined as the ratio between the maximum reflected power and the sum of all noise sources), one approach is to increase the amplitude of the reflectivity signal where noise impact should be decreased [8]. It is important for signal quality assurance to maintain a higher SNR value.

**Figure 2:**
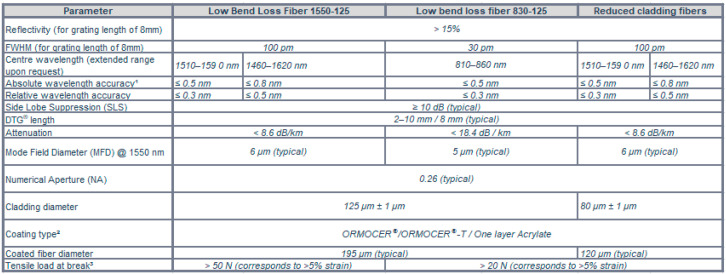
A typical data for a FBG sensor with reflectivity value.

Gratings are divided into two main types, low and high reflectivity, and are commonly available. Reflectivity values around 20% are typical for low-reflectivity sensors and are specified in the manufacturer’s data sheet for specific grating lengths. There are also very low-reflectivity FBGs available whose reflectivity value is below 5%, and an approach to obtaining optimal performance has been presented as an example in [9]. A typical technical specification of the main parameters of the FBG sensor with a given reflectivity value has been presented in Figure 2 [10].

**Figure 3:**
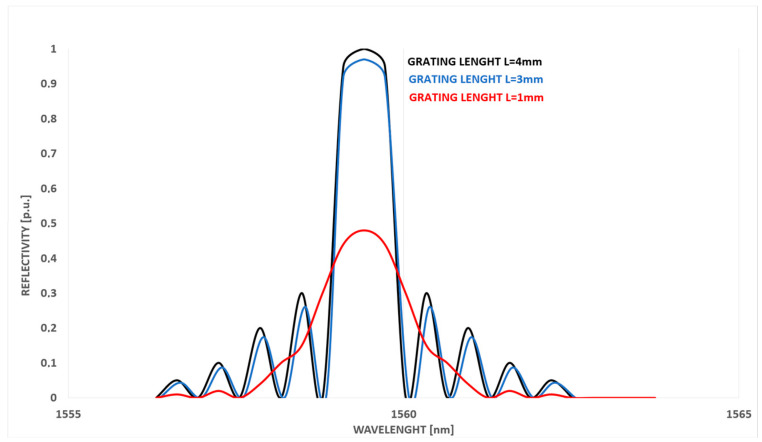
Exemplary FBG reflectivity value (R) comparison: high (R > 90%), low (R = 25%) and very low (R = 4%) reflectivity [11].

The choice of an optimal value for the reflectivity aspect depends strictly on the required application and needs to be foreseen with a specific performance of the interrogator being an integral part of the measuring system. A high reflectivity does not mean it is the most accurate or most expected to be used in every application; therefore, low-reflectivity FBGs are sometimes more suitable and in use. In most of the applications where the dynamic range of an interrogator is significantly high (e.g., >30 dB) with the use of very low reflective grating (e.g., 4%), the signal can still be measured with the SNR above the low detection limit of 10 dB, as shown in Figure 3.

It means that a dynamic interrogator system with high software gain settings for the application of optimal gain adjustment gives the opportunity to use low-reflectivity grating to reach an equal-quality signal as with high reflectivity grating but with lower gain [10].

**Figure 4:**
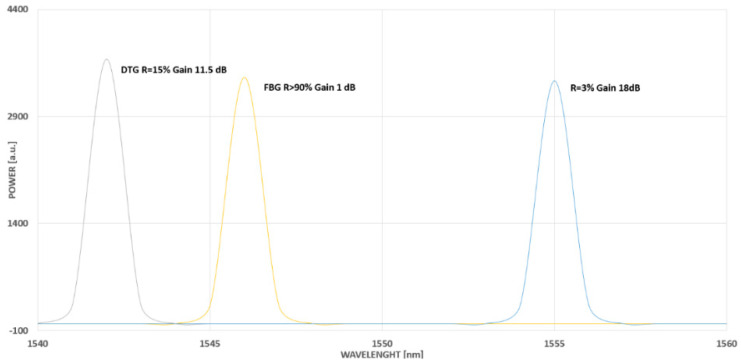
Exemplary results for FBGs with various reflectivity values—the manufacturer data [10].

As another example of various output signals, a quality signal comparison of FBG has been presented with a high (R > 90%), low-reflectivity (R = 25%), and very low-reflectivity ratio (R = 4%). The reason for that comparison has been to show the impact of gain value on signal quality and measurement stability, which can be satisfactory even with several FBGs in a single wire with even 100 sensors with low-reflectivity (<5%) applied in time domain multiplexing (Figure 3). For the sensor with a lower reflectivity, a higher gain is required (18 dB), which means more optical power for FBG wavelength detection. For a high-reflectivity sensor, a lower gain is needed (1 dB) to avoid detection of system saturation (Figure 4).

A typical structure of the fibre optic sensor has already been presented in Figure 1. One can notice the main core, cladding, and coating layers. Fibre core sizes usually vary from 4 to 50 μm and can be manufactured from a variety of available materials (like silica fibres, rare earth doped phosphate, and fluoride fibres), with refractive index to cladding being one of the main parameters to be considered during fibre type selection. An interesting type of fibre core used for increasing the photosensitivity is the germanosilicate with the germanium dopant—it impacts the core size needed in terms of aperture enlargement, which was the subject of the numerical analysis presented in [12].

The fibre cladding sizes are typically up to 125 μm. The specific interfaces exist in FBGs made of glass-polymer material types and are dependent on the fibre coating material. Such a combination may lead to unexpected coupling while the fibre bends. For that reason, to decrease the impact, the suppression of the mode coupling loss is usually introduced by controlling the photosensitivity profile [13].

**Figure 5:**
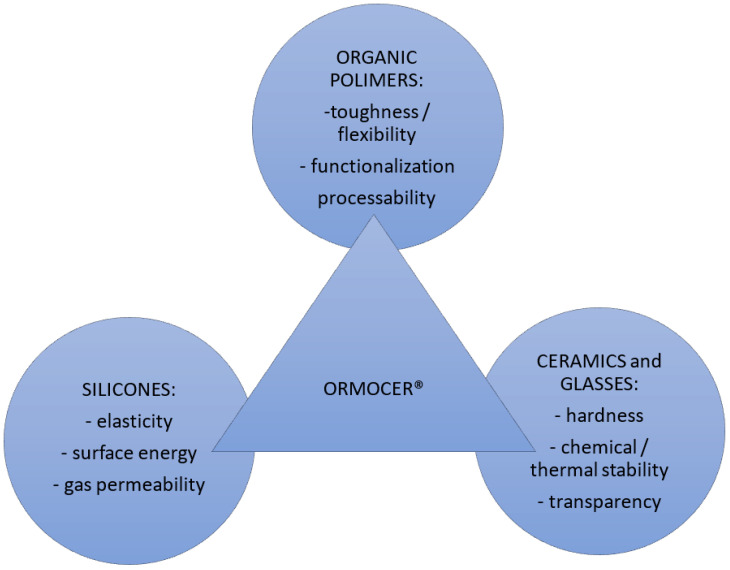
ORMOCER^®^ coating type performance [14].

Fibre coating sizes have typical sizes of around 250 μm. Polymer coatings are very common for FBG sensors and depend on required physical parameters like temperature or strain sensibility. The coating thickness and type may vary, and different options may be selected for temperature or strain sensibility. For strain sensor applications, polyamide and acrylate coatings have a temperature resistance range of up to 80 °C. It is possible to find out the specific ORMOCER and ORMOCER-T applications that are suitable for temperature ranges between −180 °C and +200 °C. The ORMOCER performance has been presented in Figure 5 [14]. This solution is also available for high-temperature applications with the use of a polyimide coating up to 300 °C and for a short time only up to 400 °C [15].

One of the most important factors in defining the reflected grating bandwidth is the Full Width Half Maximum (FWHM). The importance of this factor has been addressed since the very first research in the field of FBG sensor applications [16,17,18,19]. Nowadays, a typical value of this magnitude is 3 dB spectral amplitude below the peak of the reflectivity measured. The FWHM is expected to be at a level of about 0.1 nm bandwidth, called narrow, where 0.4 nm bandwidth is called the middle. In some cases, FBGs have this parameter of values ranging from 0.7 to 1 nm and are recognised as wide. FWHM impacts the other FBG parameters, e.g., length, refractive index, and environmental conditions like strain, temperature, and pressure, as described in [20]. The most expected are FBGs with FWHM as a narrow bandwidth.

**Figure 6:**
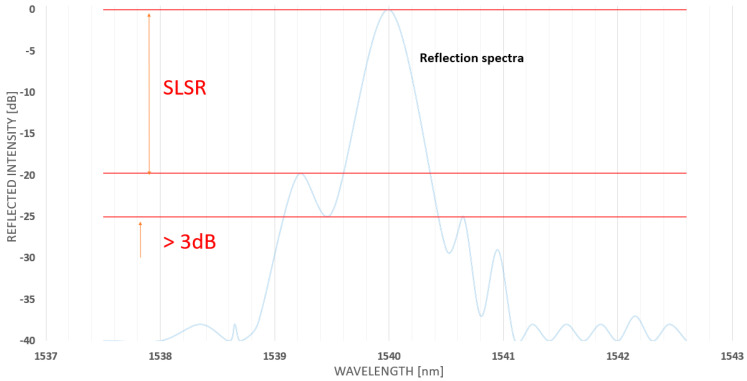
An illustration of the Side Lobe Suppression Ratio parameter.

Side Lobe Suppression Ratio (SLSR) in FBG sensors has been recognised as the area +/−3 nm around the FBG central wavelength as the second highest peak above 3 dB amplitude, and an example of it has been presented in Figure 6 [21]. SLSR is expected to be as maximum as possible and typically greater than 15 dB. Low SLSR ranges below 15 dB, and its low-reflectivity prevents FBG sensors from being used for long distances. The impact of SLSR on long-distance applications and FBG design requirements has been presented in [22].

A typical FBG sensor length is offered in the range between 4 and 8 mm for uniform FBG structures and up to 50 mm for chirped structures. Ultra-long FBG sensors (>10 cm) can reach 100 cm in length, with around 2 million periods in phase uniform type, providing high-performance fabrication [23]. It is important to avoid multiple picks in the reflected signal, as this has an impact on signal error and 3 dB bandwidth. FBG length selection has an influence on all these factors; in particular, a decrease in FBG length (e.g., from 10 mm to 5 mm and 2 mm) results in an increase in reflected bandwidth at 3 dB when no strain is applied (0 µε). However, a reflectivity reduction appears for 10 mm and 5 mm FBG lengths when strain changes from 0 µε/mm to 101.4 µε/mm, with multiple pick presence [24].

### 2.1. Operating Temperatures

Depending on the operating temperature, FBG sensors are divided into specific types. The most common are type I, type IA, type IIA, and type II. In the most general sense, type I is designed for temperatures up to +200 °C, while type II can be used in environments exposed to up to +800 °C. However, the most common operating temperature for FBG sensors is between −40 °C and +80 °C.

### 2.2. FBG Strain Transfer Characteristic

For the strain transfer analysis, the installation type, like the adhesive layer, with its thickness, width, and length, needs to be considered. Bearing in mind the long-term load applied to the host material and sensor with temperature impact as well as the shear modulus of the adhesive layer and interlayer thickness between the sensor and host material, it is necessary to remember that those are the impacting factors for strain transfer to the sensor. The adhesive layer is time-dependent, and an equation of strain transfer has been obtained. There is an example of an FBG sensor application where the strain transfer equation, including the viscoelasticity of the adhesive layer with a time-dependent factor, has been proposed to take those phenomena into account [25]. An increase in adhesive shear modulus leads to an increase in strain transfer efficiency, up to its limit. This results in a reduced influence of long-term creep on the measurement results. On the other hand, the increased adhesive length over the FBG sensor grating length leads to an increase in strain transfer efficiency and therefore may impact the shear modulus in terms of transfer efficiency, whereas the width of the adhesive layer can be neglected [26].

The thickness of the adhesive has been noted to have a significant impact on strain transfer efficiency, and it is recommended to keep it as low as possible, with optimal results in the range of 0.1–0.2 mm. The type of adhesive should also be considered; cyanoacrylate resin has been found to have better strain transfer properties than epoxy resin, considering the elastic modulus coefficient of the resin and the base material. Furthermore, the method of applying the resin to the base surface with an FBG sensor has a notable influence on the thickness and, consequently, strain transfer efficiency [27].

**Figure 7:**
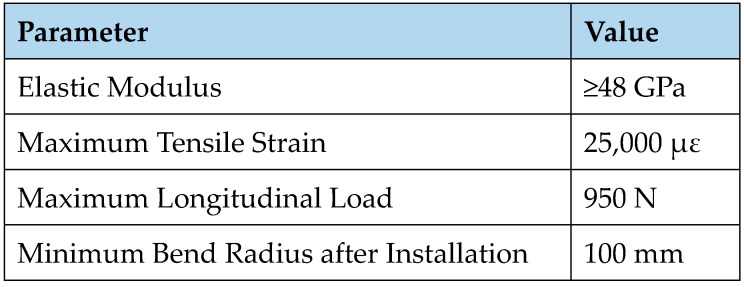
SMW-01 FBG sensor mechanical parameter table and its minimum bend radius value [29].

The power loss of an FBG fibre loop can be measured. The optical power of a fibre loop is linearly proportional to small displacements within a limited range of loop bends, where the power signal can either decrease or increase depending on the degree of bending. The mechanical specifications for the fibre, as presented in Figure 7, determine the bending radius, which in turn affects the bending stress, the fibre core reflection coefficient, and the bending loss coefficient per unit length.

The temperature-induced wavelength displacement of a Fibre Bragg Grating (FBG) (nm/°C) and the temperature-induced optical power change of a fibre loop (dBm/°C) at various temperatures must be established. Additionally, the displacement-induced wavelength displacement of an FBG (nm/μm) and the displacement-induced optical power change of a fibre loop (dBm/μm) must be determined [28].

### 2.3. Pre-Strain Requirement

There is a solution proposed where the pre-strain FBG sensors are produced to maintain the expected behaviour of the sensor when subjected to compression. For example, a pre-tension value of 3 nm has been recommended, and during installation, it has been advised to observe a decrease in wavelength as the installation progresses, such as spot welding to the base material, to minimise pre-stress losses. It is imperative to follow the FBG sensor manufacturer’s installation instructions manual, which will lead to a decrease in pre-tension, for example, of 1 nm [30].

### 2.4. Methods of Mounting FBG Sensors

The spot welding of the FBG sensor involves using a spot welder to attach the FBG to the structure. The advantages of this method include a strong bond and the ability to attach the FBG to a wide range of materials. Drawbacks of this mounting method include the potential for damage to the FBG due to the high temperatures.

Adhesive bonding as a mounting method involves applying a suitable adhesive to the FBG and then attaching it to the structure. Advantages of this method include ease of use and the ability to attach the FBG to a wide range of materials. An important drawback of this mounting method is the possibility of potential bond weakening over time.

Clamping involves using a mechanical clamp to secure the FBG to the structure. The advantages of this method include a strong bond and the ability to attach the FBG to a wide range of materials. The disadvantage of this method is that the adhesive layer can weaken over time.

A welding ring is another possible mounting method. It involves welding a ring around the FBG to fasten it to the structure. The advantages of this method include a strong bond and the ability to attach the FBG to a wide range of materials. Drawbacks include the potential for damage to the FBG due to the high operation temperatures.

An array of sensors involves attaching multiple FBGs to the structure in a specific pattern. The advantages of this method include a strong bond and the ability to attach the FBG to a wide range of materials. An important drawback is similar to that in the case of adhesive bonding.

A groove mounting method involves attaching the FBG to the structure by inserting it into a groove. The advantages of this method include ease of use and the ability to attach the FBG to a wide range of materials. Drawbacks include the potential for the bond to weaken over time.

A line method of mounting the sensors involves attaching the FBG to the structure in a linear pattern. The advantages of such an approach include ease of use and the ability to attach the FBGs to a wide range of materials. As this pattern is usually achieved with a proper adhesive, it has the same drawbacks as the one previously given.

The spiral system of FBG sensors involves attaching the FBG to the structure in a spiral pattern. Advantages of this method include ease of use and the ability to attach the FBG to a wide range of materials. Drawbacks include the potential for the bond to weaken over time. A quadrant involves attaching the FBG to the structure in a four-way pattern. Advantages of this method include ease of use and the ability to attach the FBG to a wide range of materials. Drawbacks include the potential for the bond to weaken over time.

## 3. Exemplary Numerical Model of an Industrial Boiler—Ranges of Strains and Temperatures

**Figure 8:**
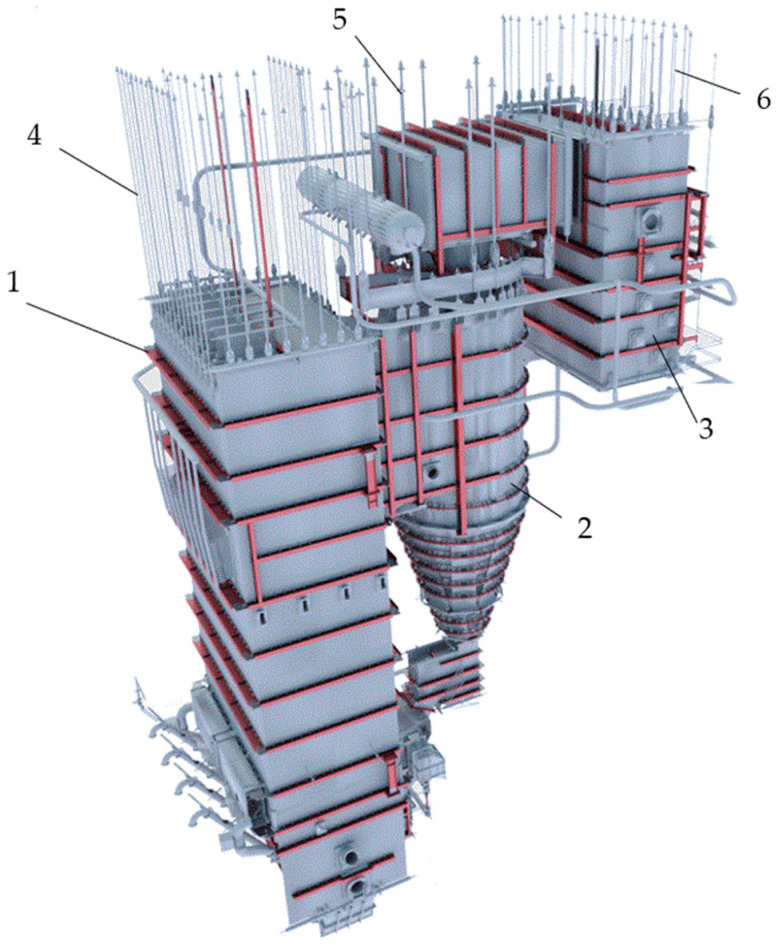
Method of suspending the CFB power boiler (pos. 1—furnace, pos. 2—separator, pos. 3—convection part, pos. 4—furnace hanger rods, pos. 5—separator hanger rods, pos. 6—convection part hanger rods).

In power boilers, typical pressure elements are inspected in accordance with applicable regulations, taking into account the operating pressure [31,32,33,34,35,36,37,38]. However, it is important to note that the actual stress on these elements is a result of all the loads they experience, including forces caused by gravity that are ‘maintained’ by hanger rods. These rods, as shown in Figure 8 [5], are responsible for suspending the furnace and other pressure parts of the boiler along with circulating fluidized bed separators. Failure to evenly distribute the weight of the boiler may result in undesirable stress and geometry changes in the pressure parts of the boiler, which in consequence may lead to a potential crack occurrence.

To prevent this black scenario, it is necessary to monitor the forces in hanger rods during the assembly stage and to perform periodic stress adjustments in combination with the geometry test of the suspension elements [4]. The operational conditions of power boilers are the subject of several factors, including the influence of internal pipelines and possible aspects at the construction phase and the limited possibility of reliable control of boiler displacements, which may possibly influence the presence of additional forces in hanger rods. Uncontrolled changes in rod forces can lead to stress concentrations, which may cause unexpected damage to the pressure parts of the boiler. Therefore, performing a periodic force measurement on hanger rods is a valuable tool for monitoring boiler loads and sheet pile stresses. Neglecting the monitoring of these forces and adjusting rods can result in unexpected shutdowns due to necessary repairs.

The hanger rods are constructed of structural carbon steel and have an operating temperature ranging from 50 °C at the top of the rod to 150 °C at the bottom. Dust levels are within acceptable limits at 30 mg/m^3^. Due to the un-machined surface of the steel rods, any sensor attachments require additional mechanical processing, such as, for example, grinding.

The temperature of the combustion gases inside the boiler can reach up to 900 °C. High temperatures are necessary to ensure an efficient incineration process and reduce harmful gas emissions. However, they can also cause damage to materials and reduce the service life of the boiler. It is therefore extremely important to monitor and control the boiler wall temperatures at various points. To avoid damage to the hanger rods when exposed to high temperatures (ranging from 300 °C to 600 °C), they are properly isolated, which allows the hanger rods to be cooled to their operating temperature between 50 °C and 150 °C. To control the rods and boiler wall temperatures, thermocouples, pyrometers, or thermal imaging cameras are used. Each of these measurement techniques has certain advantages and limitations that should be considered when choosing the right solution. However, the measurement of forces in hanger rods considering the varying temperature field remains a separate issue.

**Figure 9:**
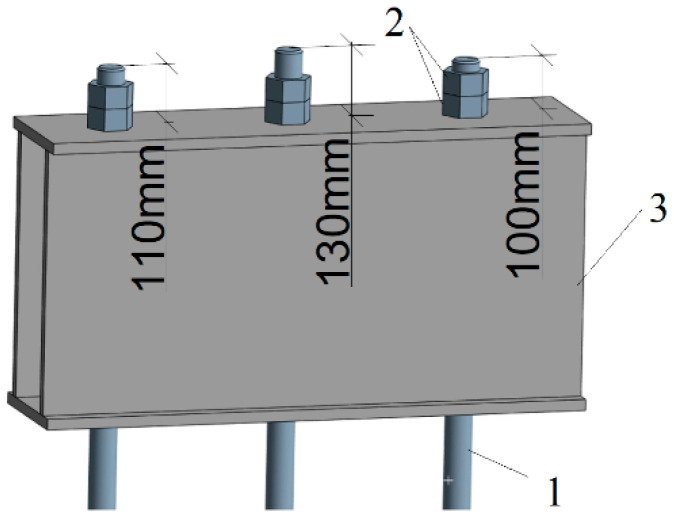
Exemplary differences in the position of boiler hanger rods (pos.1—hanger rod, pos. 2—nuts, pos. 3—construction beam).

**Figure 10:**
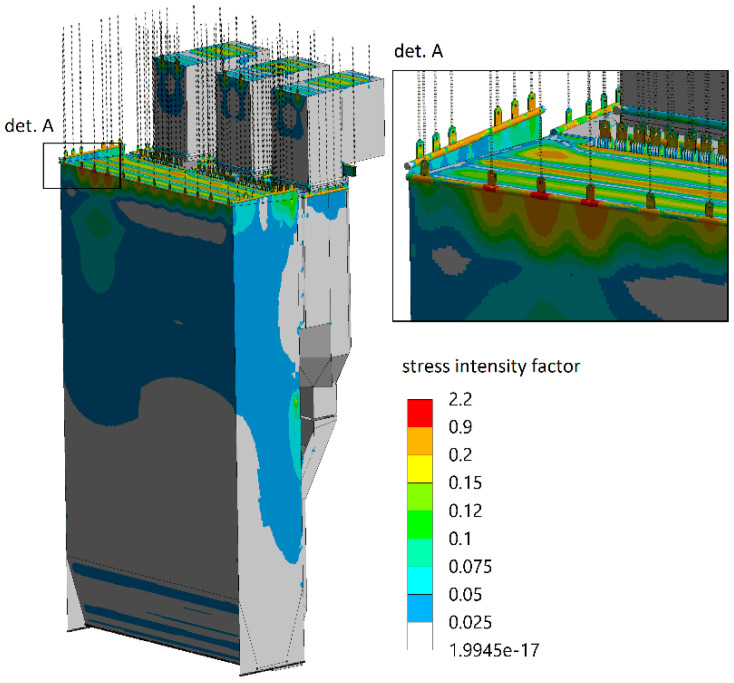
Intensity of stress in a pressure boiler in case of unbalanced force in hanger rods.

**Figure 11:**
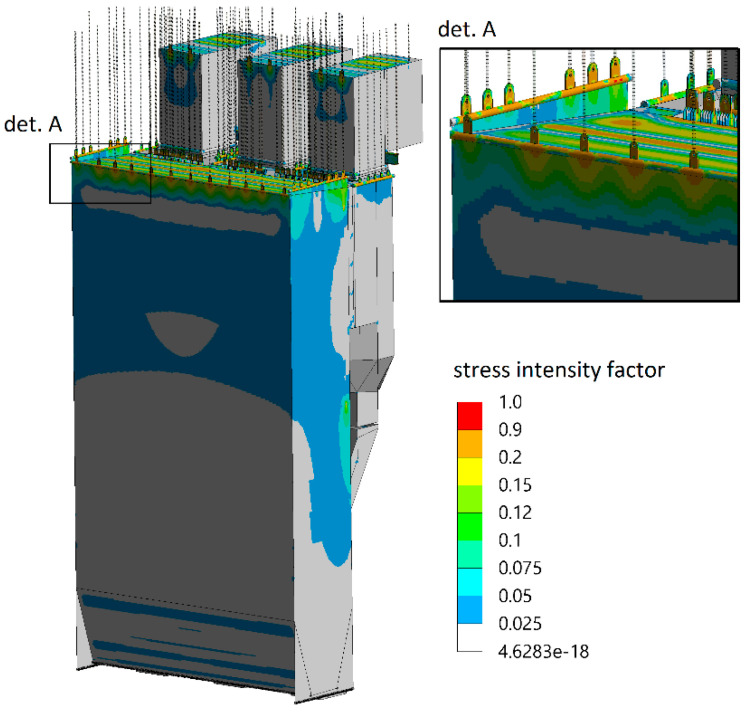
Intensity of stresses in a pressure boiler in case of optimal forces in hanger rods.

The effect of uneven load distribution on hanger rods can be verified using numerical analyses [39,40,41]. Even a slight difference in tension between individual rods (Figure 9) may result in local stress concentration areas of the supported membrane elements. As those structural parts operate in demanding conditions such as temperatures above 300 °C and high steam pressure above 10 MPa, even a small change in the tension of neighbouring rods resulting in a slight difference in the element lengths, as depicted in Figure 9, may cause an increase in the stress intensity in the pressure elements of the boiler by up to 100% (Figure 10) compared with the state when force values are adjusted and most optimal (Figure 11).

From the analysis of the industrial boiler loads shown in Figure 10 and Figure 11, it can be seen that during the operation of the exemplary boiler of 447 MWth, significant differences in the values of the stress intensity factor appear, which is a harmful phenomenon from the operational point of view. Therefore, it is extremely important to continuously monitor the state and possible changes in the forces in the rods.

**Figure 12:**
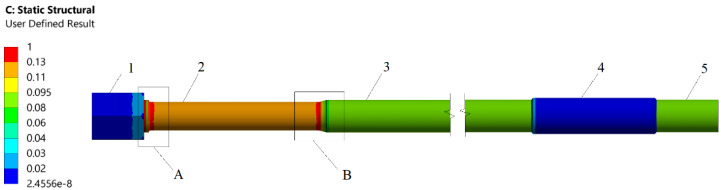
Stress concentration level in the rod: pos. 1—nuts, pos. 2—rod neck, pos. 3—cold part of the rod, pos. 4—rod connector, pos. 5—hot part of the rod, A, B—notch areas—three-axial stress.

The stress distribution along the rod is non-uniform due to its construction, and the value of allowable stresses depends on the temperature at different levels, which is a variable dependent on the boundary conditions. This is due to the connection of one end of the rod to the hot portion of the boiler, while the other end is connected to a steel beam structure that is outside the influence of flue gases and steam present in the boiler. Although the temperature of the boiler may reach as high as 400 °C, the temperature of the steel structure typically does not exceed 50 °C. Therefore, selecting a representative section of the rod to measure the forces in the rods is mandatory because it will ensure that the results are comparable to the values established through numerical analysis. The construction of the rod provides a mostly uniform state of uniaxial (tensile) stresses. Figure 12 shows the amount of stress in the rod at different levels, with a value of 1 indicating the highest stress and a value of 0 indicating the lowest. The stresses observed are primarily due to axial loading induced by the weight of the boiler, whereas thermal stresses are negligible due to the temperature gradient over a distance exceeding 10 m.

Based on the analysis results, two zones have been identified as the most suitable for measuring forces in the rod. These zones are the cold part located at position 3 and the hot part located at position 5 of the rod, as demonstrated in Figure 12. The selection of the measurement location is based on achieving a uniform state of stress. Considering sensor accessibility, the cold part of the rod (position 3 in Figure 12) is a more convenient location for measurements. The cold and hot zones are separated by the connector located at position 4, which is utilised to connect the commercial lengths of the rods. In Figure 12, zones A and B are characterised by a complex stress state resulting from the presence of a notch. The lowest temperature is observed in the part of the rod located at position 2, which also exhibits allowable stress values higher than those observed in the rest of the rod. As a result of the lack of access and shielding by the steel beam structure (pos. 3 as shown in Figure 9), it is not possible to measure forces in the neck zone located at position 2. Therefore, the optimal location for measuring the force in the rod that would be representative of the entire rod is the section marked as position 3, which is arbitrarily referred to as the cold part. The diameters of the rods at the proposed measurement location for the example boiler range between 80 mm and 100 mm. However, it should be noted that boilers are also designed with different diameters, both smaller than 80 mm and larger than 100 mm, depending on the size of the boiler and the number of rods that can be used for suspending the boiler.

The values of forces measured in several hanger rods of the boiler mentioned (and shown in Figure 10 and Figure 11) range between 76 kN and 618 kN for cold conditions (the boiler is shut down). Such force values cause the elastic rod strains to range from 7.32 × 10^−5^ mm/mm to 3.8 × 10^−4^ mm/mm. The highest measured force in the system during hot work of the boiler is 505 kN (resulting in an elastic strain of 3.2 × 10^−4^ mm/mm), while the smallest force value is 0 kN (indicating that some rods are completely loosened). Given these significant differences in force values and deformations, it is important for accuracy purposes that the sensors monitoring the forces in the rods can operate within such a wide range, as allowed by FBG strain sensor type SWS-02.

## 4. The Proposed FBG Type for the Analysed Case

**Figure 13:**
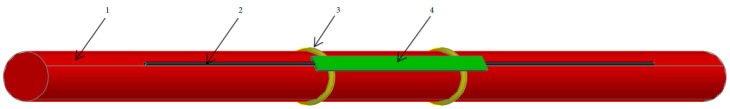
FBG sensor with clamp installation for hanger rod: pos. 1—hanger rod, pos. 2—fibre optic, pos. 3—FBG sensor clamp, pos. 4—FBG strain sensor with built in temperature sensor compensation.

Based on the analysis performed after the market inquiry as well as the requirements of the site, sensors with the following parameters have been proposed:

- Multi-mode strain and temperature FBG is to be used within the interrogator’s limited wavelength as a nanometre range when the FBG wavelength shift does not overlap the other FBG measuring range and strictly relays the sensor wavelength shift. Typical sensor grating applications include, e.g., 5 in 5 m length up to 12 FBGs in one channel.
- A free-from-strain impact reference temperature FBG sensor installed in a separate capillary,
- A sensor with a low-reflective Bragg grating,
- Careful attention to specified manufacturer requirements for fibre bends to avoid optical signal power loss,
- A system of installation that contains the clamp type—it is presented in Figure 13.

**Figure 14:**
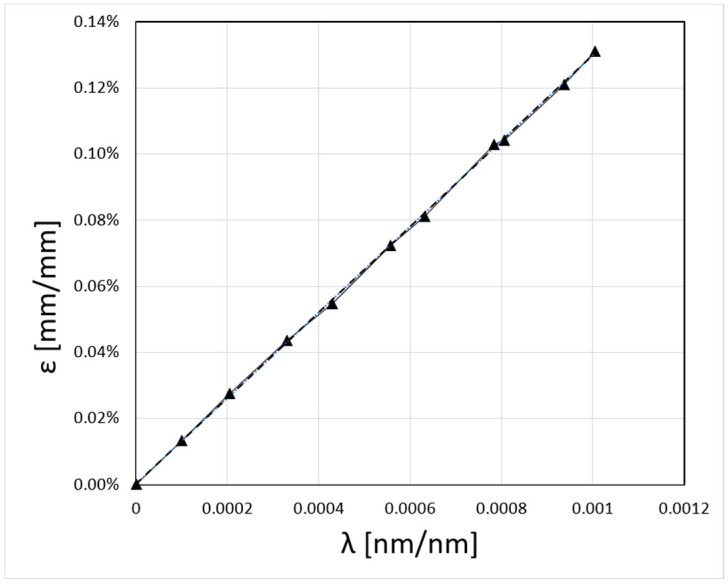
Strain ε [mm/mm] as a function of Relative Wavelenth λ [nm/nm] for the measurements with the SWS-02 sensor.

**Figure 15:**
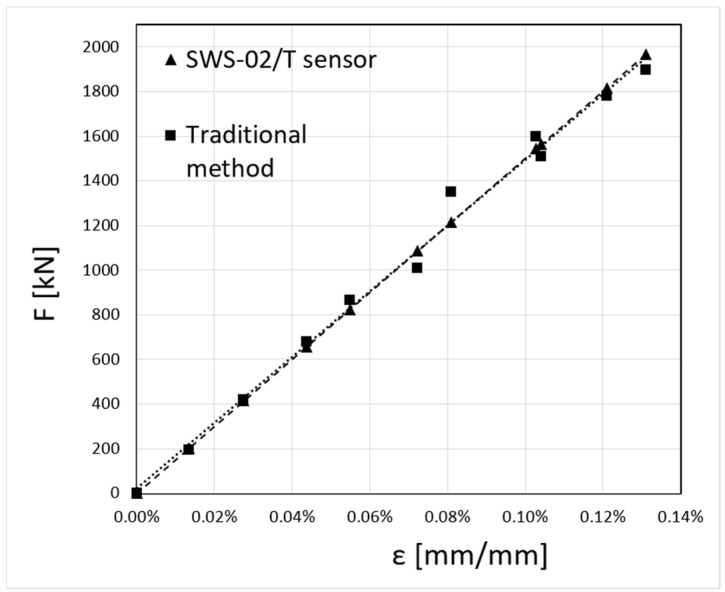
A comparison of the results of measurements of forces F [kN] as a function of strain [mm/mm] obtained using the traditional method and the method using the SWS-02 sensor.

Figure 14 shows the results of strain measurements carried out using the SWS-02 sensor manufactured by SYLEX Fibre Optics [42]. The strain magnitude is presented as a function of Relative Wavelenth λ [nm/nm]. The Full Scale (FS) accuracy of the measurements for the sensor used is 0.15%. A comparison of the measured strain values using the two methods was then performed (Figure 15). The diagram shows a comparison between the forces calculated from the strain values in Figure 14 and the forces measured using the traditional method described above, where the measurement has been carried out on a steel rod (type S355JR) with a diameter of ϕ80 mm at the surrounding temperature with the boiler switched off.

From the measurements of the axial forces in the hanger rods, shown in Figure 15, it can be concluded that there are significant differences in the measured values depending on the measurement method used. The mean absolute error (MAE) for the traditional method described earlier is 6%. This high error value is due to the inaccuracy of the conventional method of measuring the force in the bar. This method, as mentioned, involves using a special pump and actuators to pull up each rod and monitor the moment of loosening of the nut. Checking the state of the loosening of the nut is completed with a spanner or with a load cell. The illustration of the comparison of the accuracy of the measurement results summarised in Figure 15 indicates that a measurement method using fibre-optic sensors with a Bragg grating, such as the SWS-02 type [42], should be used to measure the forces in the rods, which will form the basis for further experimental studies to determine the forces in the hanger rods of power boilers.

## 5. Conclusions

This paper discusses certain technical problems that can be encountered while measuring the strain field in an environment of unusual type, which is the hanger rods of an industrial boiler. This particular application requires special care, especially in long-term sensor operations. In addition to solving the trivial problem of sensor placement, attention must also be paid to aspects such as the contact area between the sensor and the component under the test, which is essential in the case of a circular cross-section of the technical component under inspection or analysis. It should be mentioned that even the thickness of the adhesive layer as an additional interface for clamp installation can be important in terms of the effective value of the measured signal. The issue is multifaceted, and the authors hope to be able to share further results of this interesting technical application of FBG sensors soon.

Regarding the utility of the performed analysis, the FBG strain sensors are foreseen to be the best choice for constant monitoring of the force and load changes because:

- hanger rods as the main part of the steam boiler suspension system transfer only longitudinal forces, and FBG strain sensors give the opportunity to perform very precise measurements of this type of strain changes;
- the thermal stress can be easily identified by a reference temperature FBG sensor;
- harsh working environment of an industrial boiler is not a drawback for FBG sensor performance, as this type of sensor works well in such physical conditions as harsh humidity, dust pollution, or industrial pollution;
- FBG sensors are immune to electromagnetic interference, do not corrode, and do not need additional power supply;
- FBG sensors work with high precision, long-term stability, and accuracy;
- modern technical achievements allow relatively easy extension of the sensor network by the use of a proper modular interrogator;
- pre-tension equipment is available for FBG strain sensor position adjustment during installation of the final clamp;
- a wide range of measured strains in the case of rods with potentially unbalanced hanger rods is possible with the FBG strain sensor measuring system;
- it is possible to provide a constant and stable monitoring system through the use of FBG sensors, which is extremely important in this particular harsh and difficult application. Constant monitoring allows rapid reaction to any unstable situation in the analysed boiler.

## Data Availability

Not applicable.
